# The Physiological and Molecular Characterization of a Small Colony Variant of *Escherichia coli* and Its Phenotypic Rescue

**DOI:** 10.1371/journal.pone.0157578

**Published:** 2016-06-16

**Authors:** Victor Santos, Irvin Hirshfield

**Affiliations:** Department of Biological Sciences, St. John’s University, Queens, New York, United States of America; Oregon Health & Science University, UNITED STATES

## Abstract

Small colony variants (SCVs) can be defined as a naturally occurring sub-population of bacteria characterized by their reduced colony size and distinct biochemical properties. SCVs of *Staphylococcus aureus* have been studied extensively over the past two decades due to their role in recurrent human infections. However, little work has been done on SCVs of *Escherichia coli*, and this work has focused on the physiology and morphology that define these colonies of *E*. *coli*, such as small size and slow growth. *E*. *coli* strain JW0623, has a null *lipA* mutation in the lipoic acid synthase gene (*lipA*), and is a lipoic acid auxotroph. When the mutant was grown in LB medium to log phase, it showed remarkable resistance to acid (pH 3), hydrogen peroxide, heat and osmotic stress compared to its parent BW25113. Using RT-PCR and real time RT-PCR, the expression of certain genes was compared in the two strains in an attempt to create a molecular profile of *Escherichia coli* SCVs. These include genes involved in glycolysis, TCA cycle, electron transport, iron acquisition, biofilm formation and cyclopropane fatty acid synthesis. It was also demonstrated that the addition of 5 μg/ml of lipoic acid to LB medium allows for the phenotypic rescue of the mutant; reversing its slow growth, its resistance characteristics, and elevated gene expression. These results indicate that the mutation in *lipA* leads to an *E*. *coli* SCV that resembles an electron transport defective SCV of *S*. *aureus* These strains are typically auxotrophs, and are phenotypically rescued by adding the missing metabolite to rich medium. There are global shifts in gene expression which are reversible and depend on whether the auxotrophic molecule is absent or present. Looking at the *E*. *coli* SCV from an evolutionary point of view, it becomes evident that its path to survival is to express genes that confer stress resistance.

## Introduction

Bacterial strains known as small colony variants (SCVs) have been known for many years dating back to 1910 [1]. In early studies much of the work was centered on clinical specimens [1–4].

Since 1995 there has been a plethora of studies on SCVs of *Staphylococcus aureus*, particularly by Richard Proctor and those collaborating with him [1,5,6].The seminal research which connected *S*. *aureus* SCVs to persistent, recurrent antibiotic resistant mutations was published in 1995, and spurred a burst of research on the properties of these SCVs [7].

From a microbiological viewpoint the *S*. *aureus* SCVs are slow growing with a colony size quoted as being one-tenth that of a wild-type cell [1]. The *S*. *aureus* SCVs have a variety of other phenotypic changes such as decreased pigmentation, decreased hemolytic activity, and increased resistance to aminoglycoside antibiotics. An important property of the *S*. *aureus* SCVs is that they are auxotrophs, with auxotrophy for menadione or hemin being prominent [1]. The mutations which lead to the deficiency in hemin and menadione result in an inability to synthesize the heme prosthetic group of the cytochromes, and hence this leads to a defect in electron transport. This type of auxotrophic mutant has been described as an electron transport defective SCV. It was also recognized that thymidine auxotrophs lead to an SCV phenotype that was similar to that of an electron transport defective type [1]. More recently it was shown by Chatterjee et al. (2007) that thymidine auxotrophy leads to a decline in Krebs cycle activity, which results in down regulation of the electron transport machinery [8]. Of considerable importance is that addition of the missing or at least deficient metabolite leads to phenotypic rescue of the organism and reversal of the various phenotypes [1]. It should be noted that there are *S*. *aureus* SCVs which are not auxotrophic [5].

The literature on the study of *E*. *coli* SCVs is sparse in comparison to the effort made with *S*. *aureus* SCVs. In the mid-20th century there were a few publications on *E*. *coli* SCVs, but primarily on their isolation, and morphological and metabolic properties [9–12]. Colwell was able to isolate SCVs using a low concentration of 2-methyl-1, 4-napthoquinone, whereas the other investigators used copper ions at concentrations ranging from 5 x 10^−5^ M to 5 x 10^−6^ M to isolate *E*. *coli* SCVs. It was also stated that an important property of the SCVs may be lowered metabolic activity [9, 12]. *E*. *coli* SCVs have also been found associated with clinical cases [4, 13]. Roggenkamp et al. were able to isolate an *E*. *coli* SCV from a chronic prosthetic hip infection [4]. The microbe was shown to be *E*. *coli* by 16S rRNA sequencing, and was also shown to have a *hemB* gene mutation. Also Sendi et al. detected *E*. *coli* SCVs associated with prosthetic joint associated infections [13].

The *lipA* gene of *E*. *coli* encodes lipoic acid synthase needed for the synthesis of the vital enzyme cofactor lipoic acid. But even if the gene is defective, there is a salvage pathway enzyme lipoate ligase which can utilize lipoic acid in the medium [14]. Lipoic acid is an important cofactor for several enzyme systems involved in oxidative metabolism including the pyruvate dehydrogenase complex. The inactivity of this complex can result in a decline of electron transport and a concomitant decrease in the synthesis of ATP.

In this study we have chosen to analyze several classes of genes by qRT-PCR. The first group comprises *fnr* (ferric nitrate reductase), *cfa* (cyclopropane fatty acid synthase), *fecR* (iron transporter), and *wcaC* and *wcaK* (colanic acid synthesis). These genes were chosen because they are upregulated in a DNA microarray of a different *E*. *coli* SCV called IH9. All genes analyzed that involve glycolysis, TCA cycle, electron transport are based on the work of *S*. *aureus*.

In this report it is shown that the *E*. *coli* SCV *lipA* meets the criteria of an electron transport defective SCV according to the *S*. *aureus* model. The results are discussed from an evolutionary perspective.

## Materials and Methods

### Bacterial Strains

The wildtype and mutant SCV strain used in this study are listed on Table 1.

**Table 1:** *E*. *coli* K-12 strains (descendants of MG1655).

Strain	Genotype
**BW25113**	Parental strain of the *lipA* mutant *
**JW0623**	Non-functional *lipA* gene **

* *E*. *coli* Genetic Stock Center, Yale University

** Keio Collection from *E*.*coli* Genetic Stock Center, Yale University

### Media and bacterial culturing techniques

The wild type strain used in this study is BW25113 and the mutant used is JW0623, also referred to as *lipA*, which is a small colony variant. It was created by insertion mutagenesis that resulted in a disruption of function of the enzyme lipoate synthase and conferred kanamycin resistance to JW0623. The *lipA* mutation was verified by DNA sequencing.

Both strains (BW25113 and *lipA)* were cultured in Luria Bertani (LB) medium to log phase. When needed, lipoic acid was added as a supplement at 5μg/ml. The incubation of both cultures was done in a New Brunswick Scientific Environmental gyratory incubator at 37°C, with shaking at 200 RPM. Stock cultures of both strains were kept at 4°C in LB broth, and sub-cultured also on LB agar plates and maintained at 4°C. Growth of cultures was monitored with a Carolina Spectrophotometer model 65–3303 at 580 nm; the growth experiments were run in triplicate.

Microsoft office excel 2013 Software was used to generate a growth curve by plotting the OD versus time in minutes. In all experiments, the WT cells were grown to an OD of 0.60 and the mutant to 0.30 OD. This is log phase for both strains and CFU determination showed that the number of cells is the same, ca. 1 x 10^8^ cells/ml.

In order to compare colony size on LB agar plates, the *lipA* strain was grown to log phase in LB medium and streaked on LB plates with and without 5μg/ml lipoic acid. These plates were inoculated at 37°C for 20h.

### Survival assay using inorganic acid (LB, pH 3.0)

In order to compare the percentage survival of the WT and mutant incubated in the presence of acid pH 3.0, a survival assay was done. Both the WT and mutant were grown to log phase using the shaker/incubator at 37°C.

Once log phase in LB medium was reached, the initial CFU was determined by performing an appropriate serial dilution in 0.85% saline and plating out in duplicate 100 μl of the 10^−5^ and 10^−6^ dilutions. The plates were incubated overnight at 37°C, and the colonies were counted the following day to establish a baseline of the number of colonies present before the strains were subjected to the lethal acid shock. The initial number of CFUs was always ca. 1 x 10^8^ cells/ml.

100 μl of the WT or mutant cells were then transferred to 900 μl of Luria Bertani medium/ pH 3.0. The cells were incubated for thirty minutes in a 37°C water bath without shaking. Thereafter, the cells were diluted appropriately in 0.85% saline and spread plated on LB agar plates. The plates were incubated overnight at 37°C and the colonies were counted the following day. In all survival experiments, the cells were normalized to 1 x 10^8^ CFU/ml. The percent survival = CFU survival/CFU original x 100.

Microsoft excel 2013 was used to generate a graph showing the percentage survival of both the WT and the *lipA* mutant.

### Survival assay using 10 mM hydrogen peroxide

In order to compare the percentage survival of the WT and mutant incubated in the presence of 10 mM hydrogen peroxide a survival assay was done. Both the WT and mutant were grown to log phase in LB using the shaker/incubator at 37°C. The procedure was the same as for the acid survival experiments but with 10 mM hydrogen peroxide in neutral LB medium. The incubation was also 30 min.

Microsoft excel 2013 was used to generate a graph showing the percentage survival of both the WT and the *lipA* mutant.

### Survival assay using inorganic acid (LB, pH 3.0) or 10 mM hydrogen peroxide on *lipA* log phase cells grown in the presence of 5μg/ml lipoic acid

In order to determine if the mutant reverses to WT characteristics after exposure to lipoic acid, survival assays were done using pH 3.0 acid medium or 10 mM hydrogen peroxide to allow us to compare how the cells survive with and without the lipoic acid.

The procedure was the same as above, but the *lipA* cells were grown in the presence of 5μg/ml lipoic acid.

### Survival assay using 51°C as a stress factor

In order to compare the percentage survival of the WT and mutant incubated at 51°C a survival assay was done. Both the WT and mutant were grown to log phase in LB medium using the shaker/incubator at 37°C. The procedure was done as above for acid and hydrogen peroxide but using 51°C in neutral LB as the shock agent for 30 mins.

Microsoft excel 2013 was used to generate a graph showing the percentage survival of both the WT and the *lipA* mutant.

### Survival assay to compare percent survival of *lipA* and WT log phase cells incubated in the presence of 8.0% sodium chloride

The mutant cells and WT cells were grown in LB medium in a shaker/incubator at 37°C to log phase. The procedure was done as above for acid, hydrogen peroxide, and heat but using 8.0% sodium chloride in neutral LB as the shock agent for 3 hrs.

### Cyclopropane fatty acid (CFA) whole cell lipid analysis of log phase cells

In order to analyze the lipid content of both the WT and *lipA* mutant in log phase, cells were grown in a shaker/incubator at 37°C. A 100 ml volume of LB medium was used to grow the mutant cells to log phase, and a 50 ml volume was used to grow the WT to log phase.

After the desired OD was reached, the cells were poured into 50 ml sterile conical tubes, and centrifuged for ten minutes at 8,000 RPM in a JA-12 rotor at 4°C using the Beckman Coulter Centrifuge. After 10 minutes, the supernatant was discarded, and 10 ml of 10mM Tris-HCl buffer (pH 7.4) was added to each tube. The cells were washed twice using this buffer for 10 minutes at 8,000 RPM.

After the second wash, the supernatant was discarded and each strain was then suspended in 5.0 ml of the buffer.

The cell suspensions were transferred to a 65 or 75 ml pear-shaped sterile flask and quick frozen in dry ice acetone. They were then stored at -20°C until the following day when they were lyophilized using a Virtis-Lyophilizer, after which the samples were removed and stored at -20°C. Ten to 15 mg of each sample was shipped to Microbial I.D. (Newark, DE) for whole cell lipid analysis using the Sherlock gas chromatography system.

### DNA extraction

For primer verification purposes, genomic DNA was extracted from *lipA* and BW25113 using a Sigma-Aldrich ^®^ Bacterial Genomic Miniprep Kit. DNA was eluted with buffer and quantified spectrophotometrically for quality and quantity. All samples had concentrations greater than 200 ng/μl and 260/280 ratios ranging from 1.80–2.00.

### RNA extraction from the WT strain and the mutant *lipA*

Both the WT and the mutant strain were grown at 37°C in a rotary/incubator to log phase for the purpose of RNA extraction. The protocol from the kit supplied by RiboPure-Bacteria was used to extract the messenger RNA.

A digital UV spectrophotometer was used then to quantify the RNA and to determine the A_260_/A_280_ ratio. After extracting the RNA, the concentration of total RNA extracted was determined to be 800ng/μl and the A_260_/A_280_ was determined to be 1.9. The RNA was aliquoted to eppendorf tubes to a final concentration of 2ng/μl to use for the RT-PCR reactions and 100ng/μl to use for the qRT-PCR reactions.

### RT-PCR procedure to investigate the expression of selected glycolysis genes and biofilm genes in both the WT and the mutant grown in the absence and presence of 5μg/ml lipoic acid

After the concentration of the RNA was established, the protocol from Qiagen one-step RT-PCR kit was followed to perform the reverse transcriptase-polymerase chain reactions. A total of 2ng/reaction of total RNA was used. The total volume of each reaction was 25μl. Electrophoretic analysis was done using a 1.0% TBE agarose gel for 30 mins at 100 volts. The New England Biolabs 2-Log DNA ladder was used to estimate the size and intensity of the bands.

### Gene expression analysis using real time RT-PCR

After the concentration of the RNA was established, the protocol from Bio-Rad iTaq^TM^ Universal SYBR^®^ Green One-Step Kit was followed with some modifications to perform the expression analysis on selected genes. The genes analyzed for differential expression were *fnr*, *fecR*, *cfa*, *wcaC*, *wcaK*, *pykF*, *pgi*, *pgk*, *acnB*, *sdhC*, and *cyoA*.

A total of 100 ng/reaction was used in each experiment carried out. The house keeping gene used in this study was the 16S rRNA gene. Table 2 lists gene names and all primers used in this investigation.

**Table 2:** Gene name and primers used in this investigation.

Gene	Sequence 5´ to 3´
***Eno*, Enolase**	**for TAACCCGACTGTTGAAGCC**
	**rev AGTCAGATTCGTCCAGACC**
***fbaA* Aldolase**	**for TACTGACTCCATCAACGCC**
	**rev TAGTTCAGAACGCCTTCCC**
***Pgi*, Phosphoglucose isomerse**	**for TTCCACCAATGCCAAAGCC**
	**rev TGTTTACCCAGTTCCACGCC**
***Pgk*, Phosphoglycerate kinase**	**for TTCAACAAAGGCGAGAAGAAAG**
	**rev TGTCAGCAATGCCGAACAG**
***pykF*, Pyruvate kinase I**	**for CGAAAACATCCACATCATCTCC**
	**rev AACGCCTTTGCTCAGTACC**
***sdhC*, Succinate dehydrogenase**	**for CGGTGTGATCACCTTTGTTG**
	**rev AGGACTCCTGCGAGAAGTGA**
***acnB*, Aconitase**	**for AAGTGCGTAACCACGAAACC**
	**rev CAAAGGCCTGCTCAACTTTC**
***cyoA*, Cytochrome o oxidase**	**for TTGCAGGCACTGTATTGCTC**
	**rev CATGCCTTCCATACCTTCGT**
***wcaC*, Colanic acid biosynthesis gene**	**for GTTTATTTCCCCCAGCCAG**
	**rev TGACATACTCCTCCAGCATC**
***wcaK*, Colanic acid biosynthesis gene**	**for AAAAAAGTCCTCCGTCGCC**
	**rev CCTTCCTCCATACCCCTTTC**
***fecR*, Ferric citrate transporter**	**for GCAACAGTGGTATGAACAGG**
	**rev GGTATTTTTCAGCGGGAACG**
***cfa*, Cyclopropane fatty acid synthase**	**for ACCAACTCCCCCATCATTTC**
	**rev TCATACCACGCCATCAACG**
***Fnr*, Fumarate nitrate reductase**	**for ATGATCCCGGAAAAGCGA**
	**rev TCAGGCAACGTTACGCGT**
***16s rDNA*, 16S ribosomal RNA**	**for TTACCCGCAGAAGAAGCACC**
	**rev ACATTTCACAACACGAGCTGAC**

The genes *fnr* (fumarate nitrate reductase), *fecR* (ferric citrate transporter), *cfa* (cyclopropane fatty acid synthase), and *wcaC* and *wcaK* (colanic acid biosynthesis) were chosen because they were found to be upregulated in a DNA microarray of another *E*. *coli* SCV isolated in this lab (unpublished results). The initial goal here is to determine if they are also upregulated in the *lipA* SCV as this would suggest that these genes could serve as biomarkers for *E*. *coli* SCVs. The other genes were chosen on the basis of studies with *S*. *aureus* SCVs.

### Statistical Analysis

Statistical analysis of data was performed using the Microsoft excel ANOVA software.

## Results

### The growth of the WT and the mutant strain with and without 5μg/ml lipoic acid in LB medium

**Fig 1:**
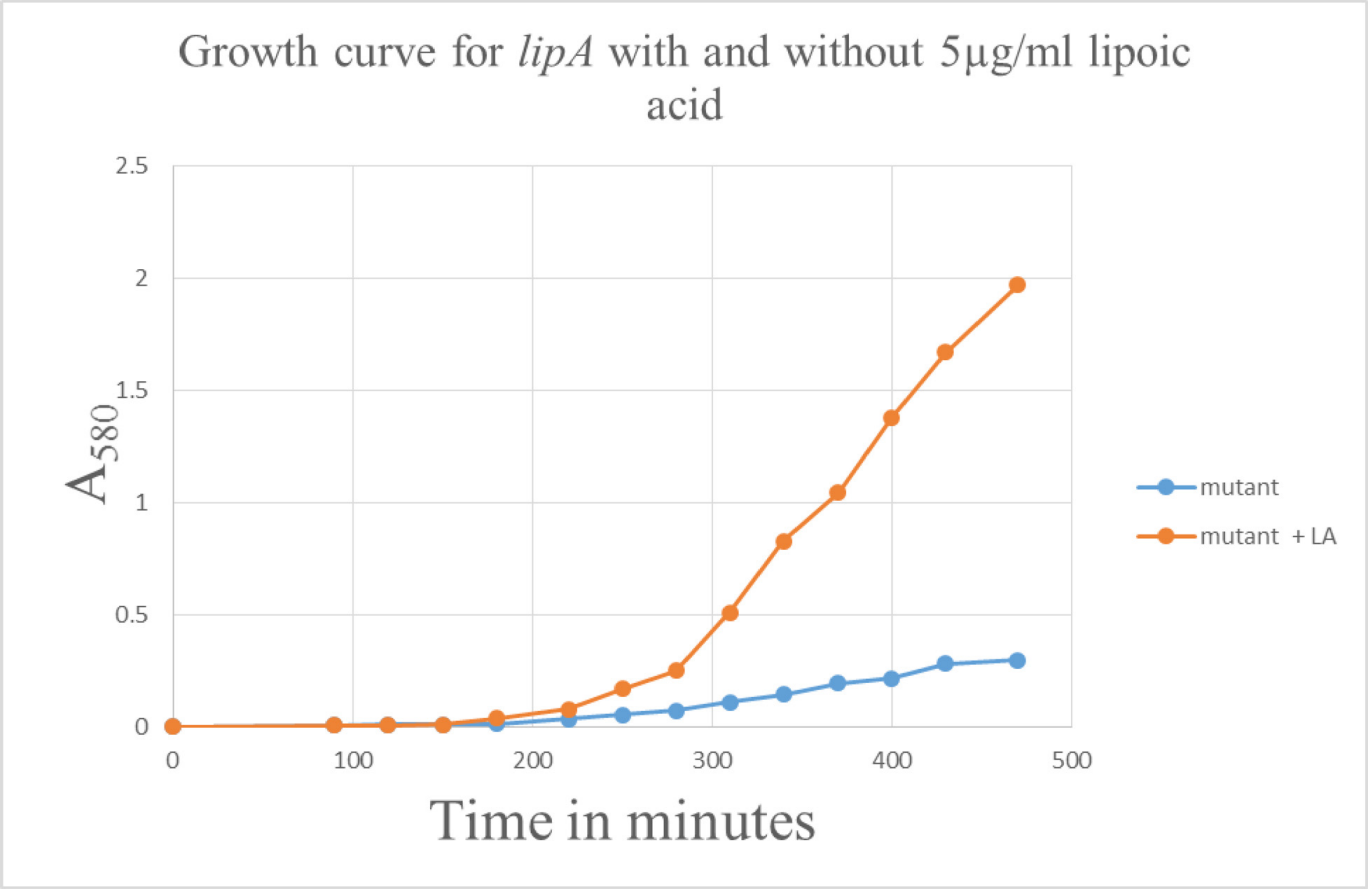
Growth curve for *lipA* cells grown with and without lipoic acid (5μg/ml).

The generation time of the WT on LB medium was 25 minutes. With the *lipA* mutant, the generation time was 64 minutes in LB alone, but accelerated to 25 minutes when supplemented with 5μg/ml lipoic acid. The morphological characteristics of the mutant on an agar plate are smooth, convex, glossy, and off white in color as observed by others [12]. But with the supplementation of lipoic acid, the colonies appear larger and beige in coloration resembling the WT. It is clear from this experiment that lipoic acid is indeed critical for the optimal growth of the mutant (Fig 1). The SCV *lipA* is auxotrophic for lipoic acid, analogous to results found with *S*. *aureus* SCVs which lack menadione, hemin, or thymidine [1].

**Fig 2:**
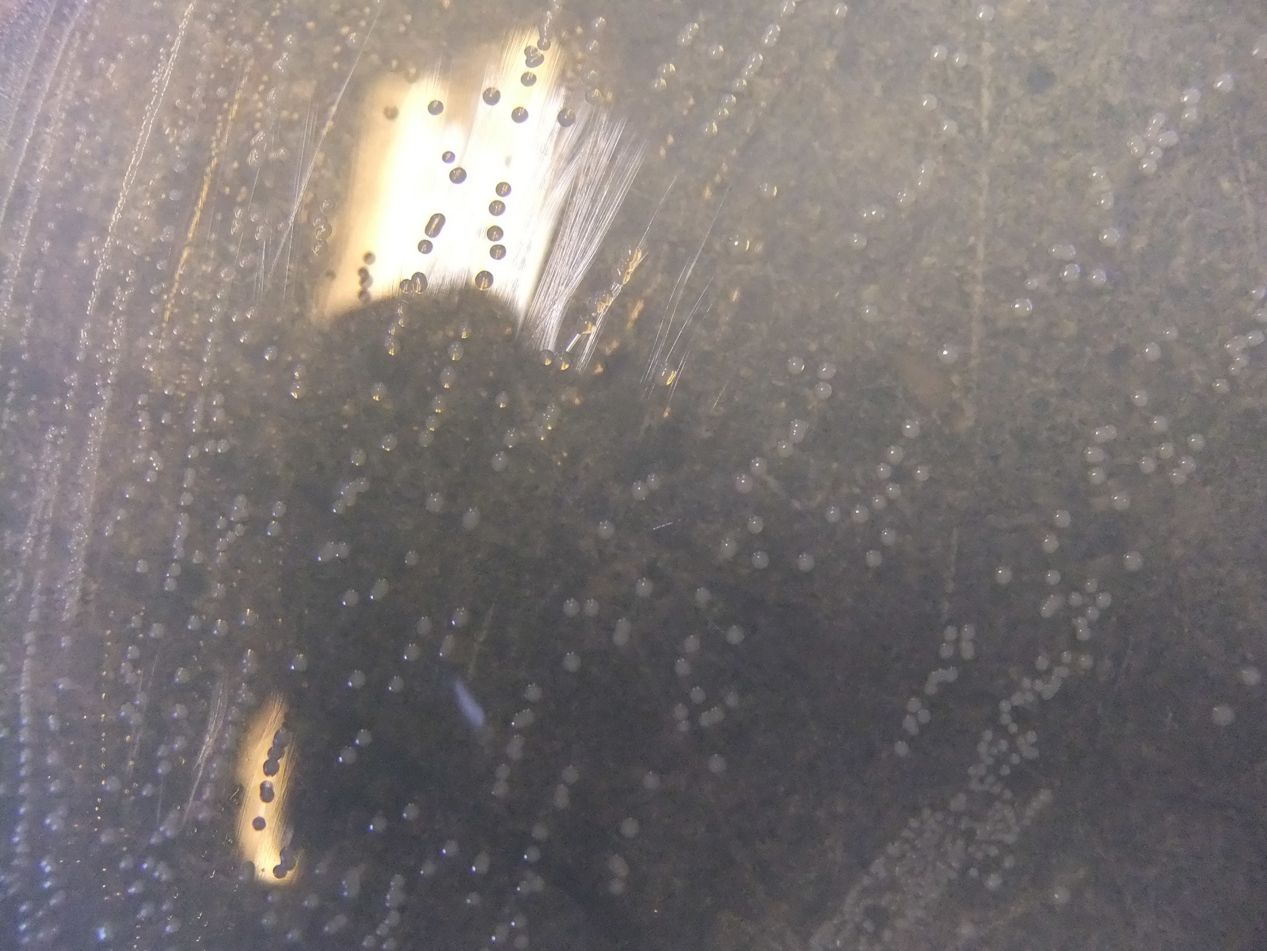
*lipA* cells plated on LB alone.

**Fig 3:**
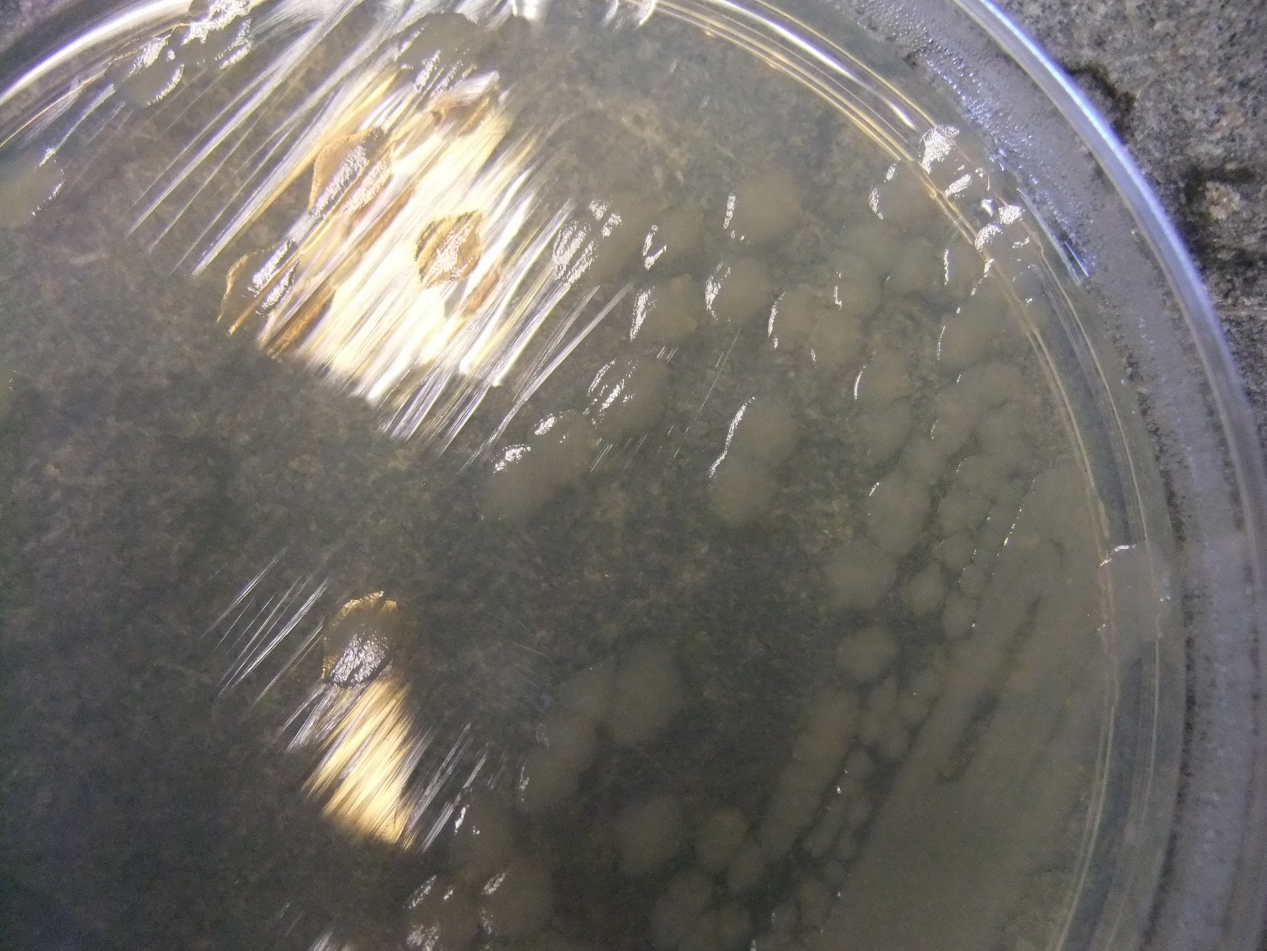
*lipA* grown on LB agar plate supplemented with lipoic acid (5μg/ml).

When grown on the LB agar plate supplemented with lipoic acid (5μg/ml), the mutant *lipA* colonies appear much bigger in size and similar to the wild type in coloration and morphological attributes (Figs 2 and 3).

### Survival assay using inorganic acid (HCl, pH 3.0) on log phase cells of WT, *lipA*, and *lipA* grown with lipoic acid

After counting the CFUs and normalizing the cell count, it was clear that the survival of the *lipA* mutant is much higher than the WT in the presence of inorganic acid at pH 3.0 (LB, pH 3.0). The survival of the *lipA* mutant was determined to be 4.3% as opposed to 0.002% observed for the WT. On average the mutant exhibits 2,150 times better survival than the WT.

**Fig 4:**
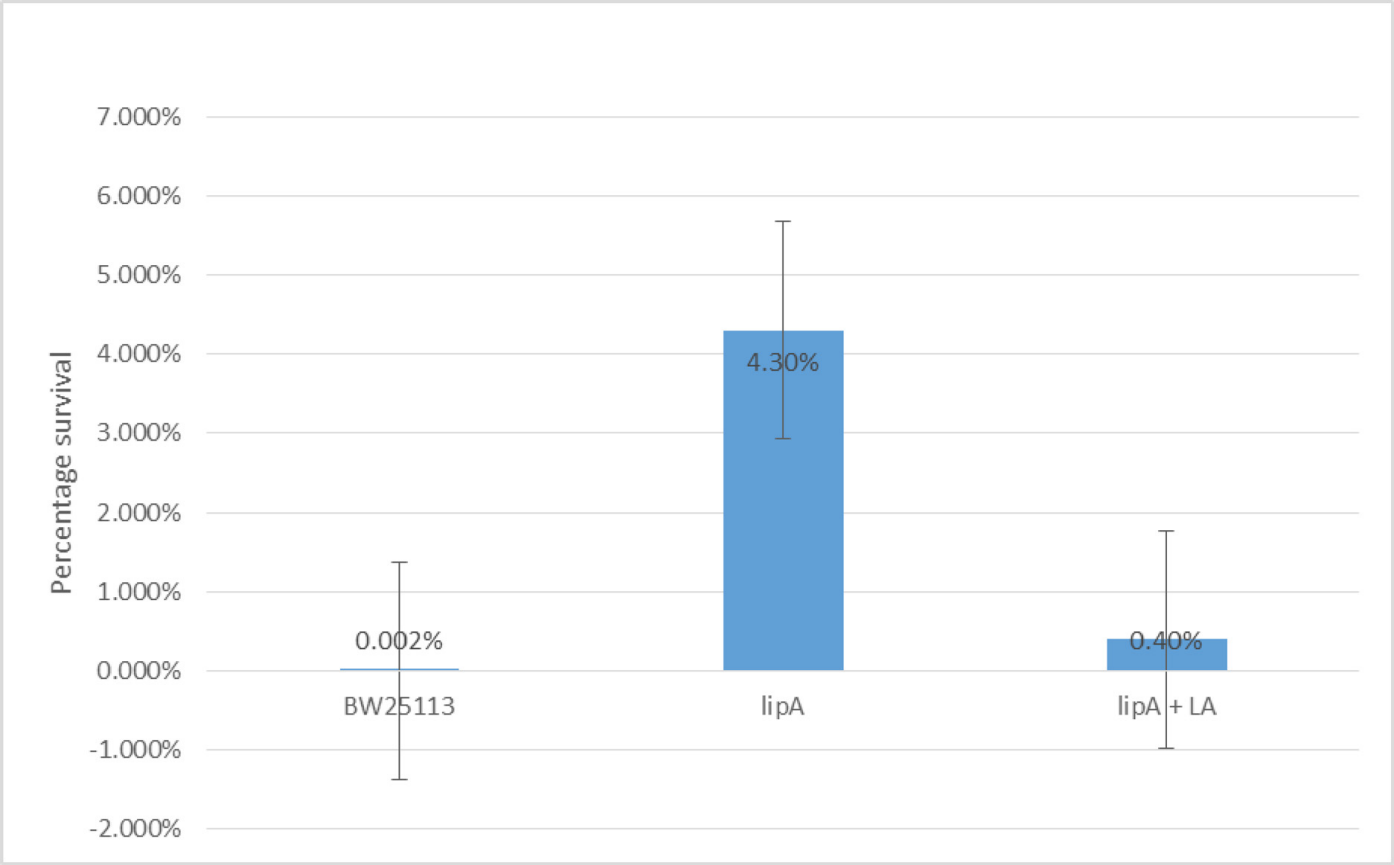
Percentage survival of WT, *lipA*, and *lipA* grown with 5μg/ml lipoic acid after inorganic acid exposure. p<0.05

Mutant cells that were grown in the presence of 5μg/ml lipoic acid to log phase and then exposed to acid for 30 minutes displayed very little resistance to the acid. (Fig 4).

### Survival assay using 10 mM hydrogen peroxide on log phase cells of WT, *lipA*, and *lipA* grown with lipoic acid

After counting the CFUs and normalizing the cell count, it was clear that the survival of the *lipA* mutant is dramatically higher than the WT in the presence of 10mM hydrogen peroxide. The survival of the *lipA* mutant was observed to be 69% as opposed to 0.02% for the WT. On average the mutant exhibits 3, 450 times better survival than the WT.

**Fig 5:**
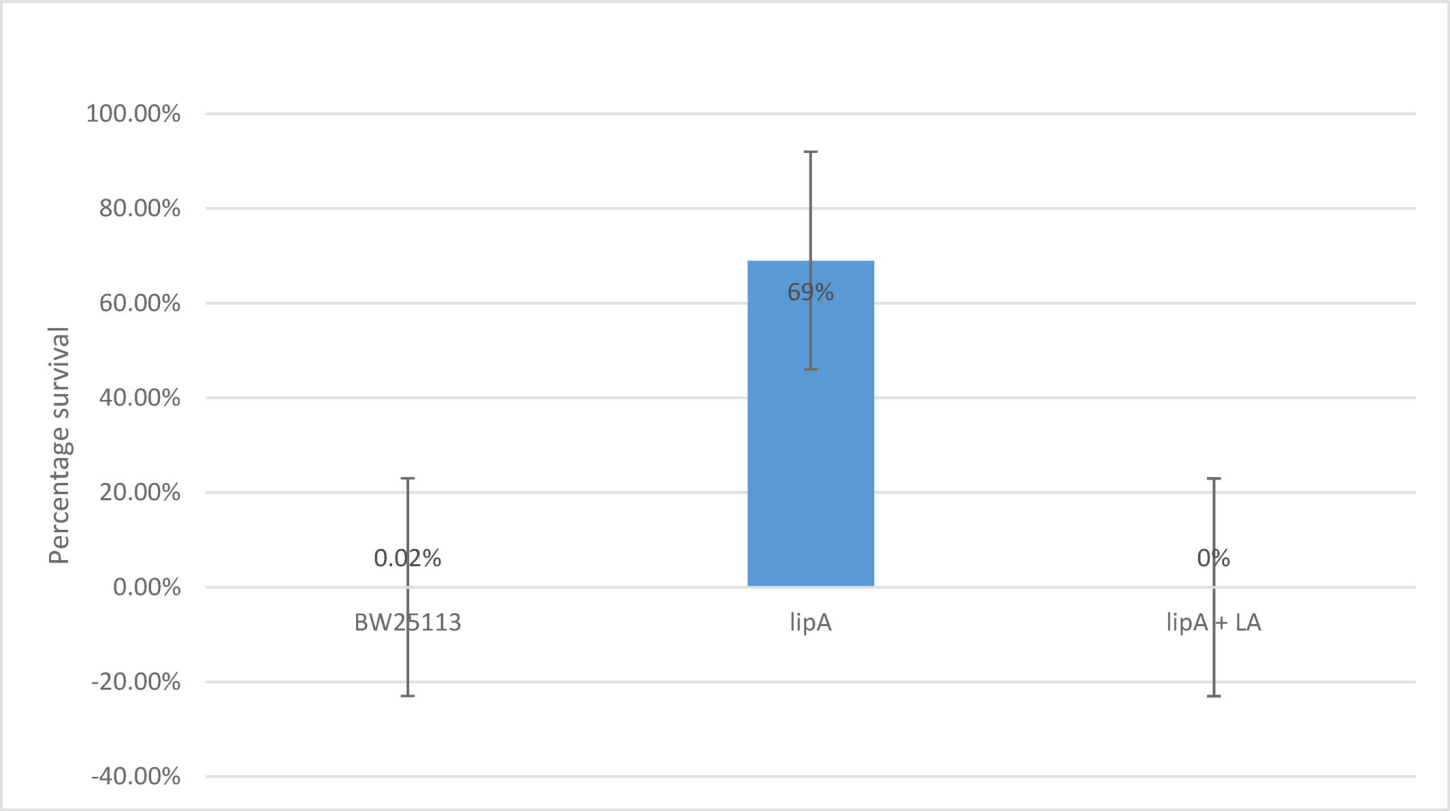
Percentage survival of WT, *lipA*, and *lipA* cells grown with/without lipoic acid after 10 mM hydrogen peroxide exposure. p<0.05

Mutant cells that were grown in the presence of 5μg/ml lipoic acid to log phase and then exposed to 10 mM hydrogen peroxide displayed very little resistance to the stress factor. 0% survival was observed. Thus, the mutant loses the ability to resist hydrogen peroxide if grown in the presence of this concentration of lipoic acid. It reverses back to its wild type characteristic (Fig 5).

### Survival assay at 51°C using log phase cells

**Fig 6:**
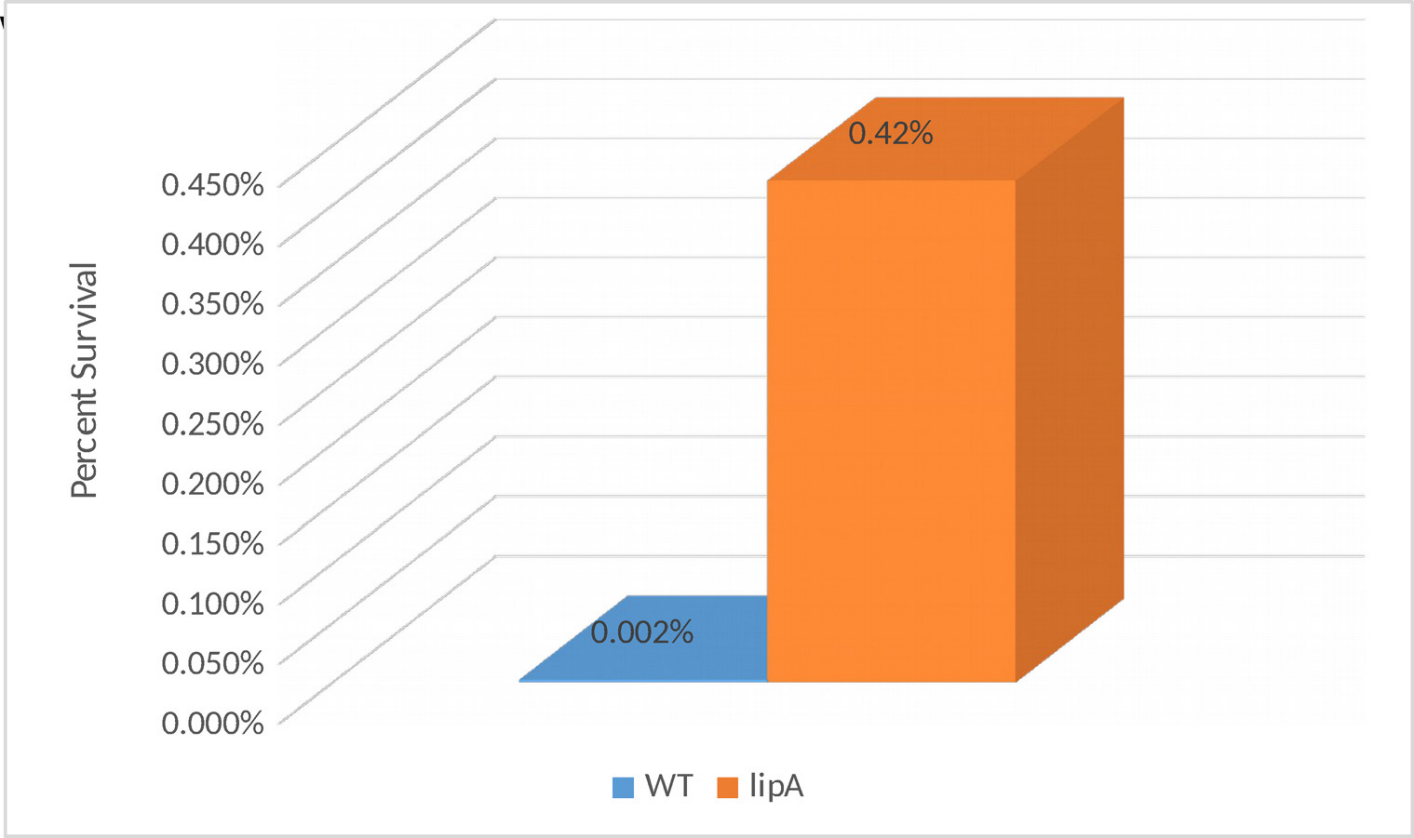
Percentage survival of log WT and mutant incubated in LB for 30 minutes at 51°C. p<0.05

After counting the CFUs and normalizing the cell count it was clear that the survival of the *lipA* mutant is substantially higher than the WT when incubated at 51°C. On average, the mutant exhibits 210 times better survival than the WT (Fig 6).

### Survival assay comparing the percent survival of WT and *lipA* log phase cells exposed to 8.0% sodium chloride

**Fig 7:**
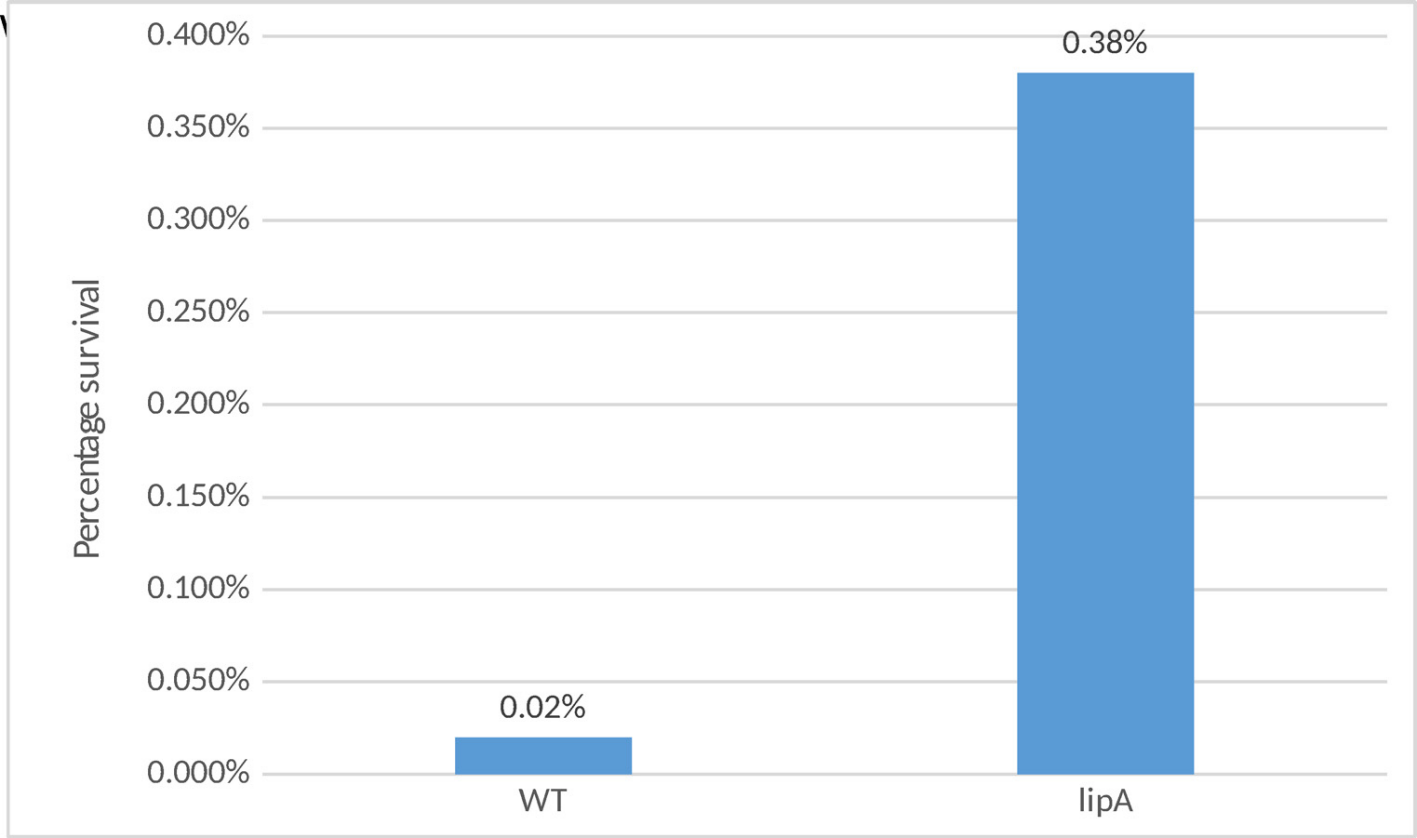
Percentage survival for log phase WT and *lipA* cells incubated with 8.0% sodium chloride. p<0.05

The cell count for both the WT and mutant was normalized to 1 x 10 ^8^ CFU/ml. After counting the CFUs and normalizing the cell count it was clear that the survival of the *lipA* mutant is higher than the WT when incubated in the presence of 8.0% sodium chloride. On average, the mutant exhibits 18 times better survival than the WT (Fig 7).

### Cyclopropane fatty acid content upon whole cell lipid analysis of log phase cells of WT, *lipA*, and *lipA* grown with lipoic acid

The samples of WT and mutant freeze-dried cells were sent to Microbial I.D. for whole cell lipid analysis. Most important were the concentrations of the C17 and C19 cyclopropane fatty acids. Based on the results, the mutant had a decidedly higher CFA content than the wild type in log phase. The C17 CFA content of the SCV was 6.7%, but the only 2.2% in the WT. Correspondingly, the C19 CFA content was 0.82% and 0.23%. All experiments were done at least in duplicate.

**Fig 8:**
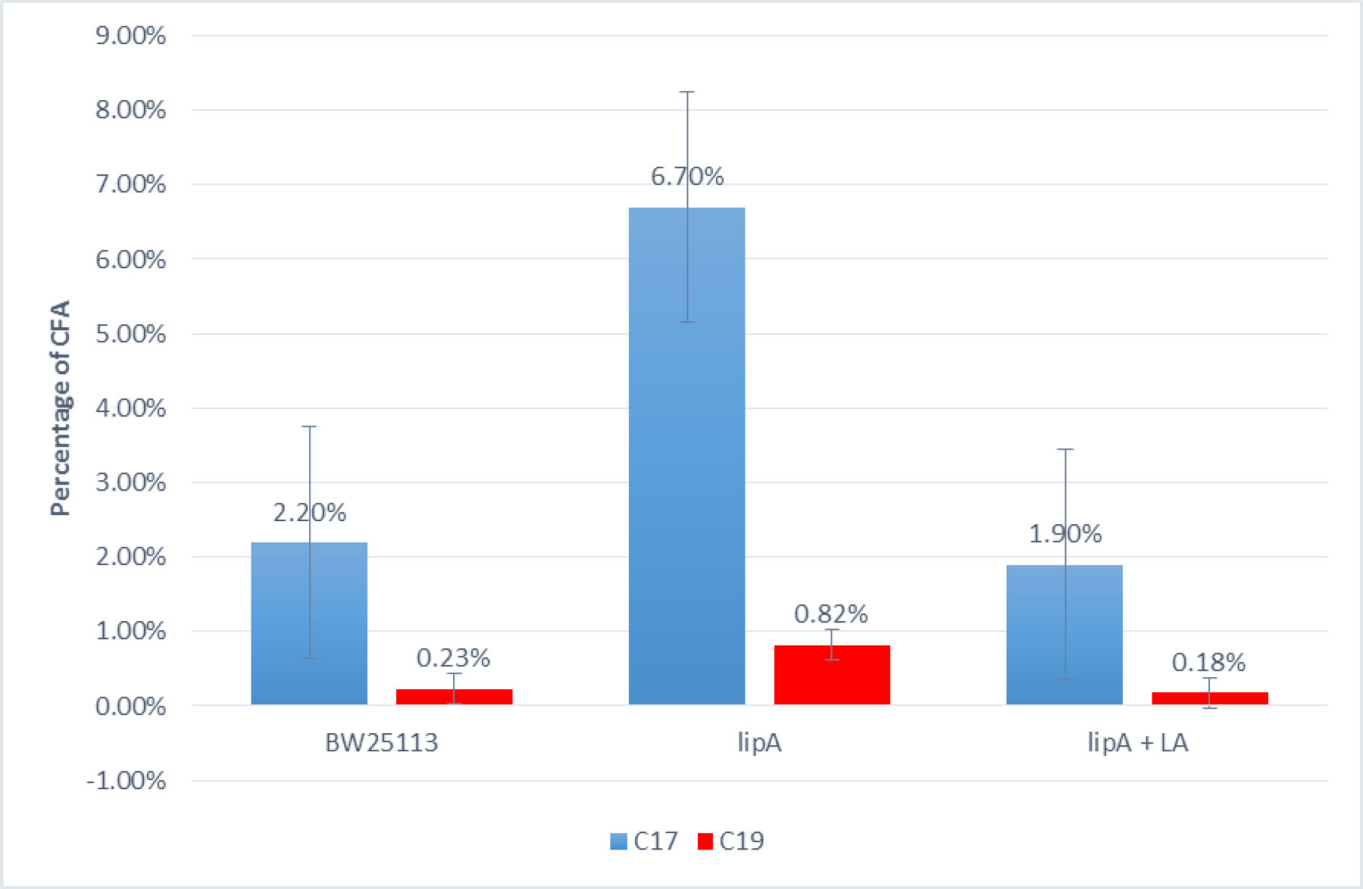
CFA content of WT, *lipA*, and *lipA* grown in the presence of 5μg/ml lipoic acid to log phase. p<0.05

When grown in LB with lipoic acid supplement (5μg/ml), the CFA content readjusted in the SCV to the WT level being 1.9% for the C17 CFA and 0.18% for the C19 CFA (Fig 8).

### RT-PCR analysis of selected glycolysis genes in WT, *lipA* mutant grown in LB medium and the *lipA* mutant grown in the presence of 5μg/ml lipoic acid

After running the electrophoresis gel and analyzing it with the bio-illuminator, the expression of four of the five genes analyzed was found to be elevated in the mutant as compared to the wild type. Based on the RT-PCR gel, the expression of aldolase (*fbaA*) was about equal in the two strains. Enolase (*eno*), pyruvate kinase (*pykF*), phosphoglycerate kinase (*pgk*), and phosphoglucose isomerase (*pgi*) were expressed more highly in the mutant.

The expression of *pykF* and *pgk* in the mutant grown in the presence of lipoic acid was observed to be lower than their expression in SCV cells grown only in LB medium and similar to the WT level (S1–S3 Figs).

### RT-PCR analysis of *wcaC* and *wcaK* genes in WT and the *lipA* mutant grown in LB medium and the *lipA* mutant grown in the presence of 5μg/ml lipoic acid

After running the electrophoresis gel and imaging the gel with the bio-illuminator, the expression of both *wcaC* and *wcaK* were found to be higher in the mutant as compared to the WT; whereas the expression of both the *wcaK* and *wcaC* genes in the mutant, when grown in the presence of lipoic acid, were observed to be lower than their expression in *lipA* grown only in LB medium, and similar to the level in WT (S4–S6 Fig).

### Gene Expression analysis using real time RT-PCR

**Fig 9:**
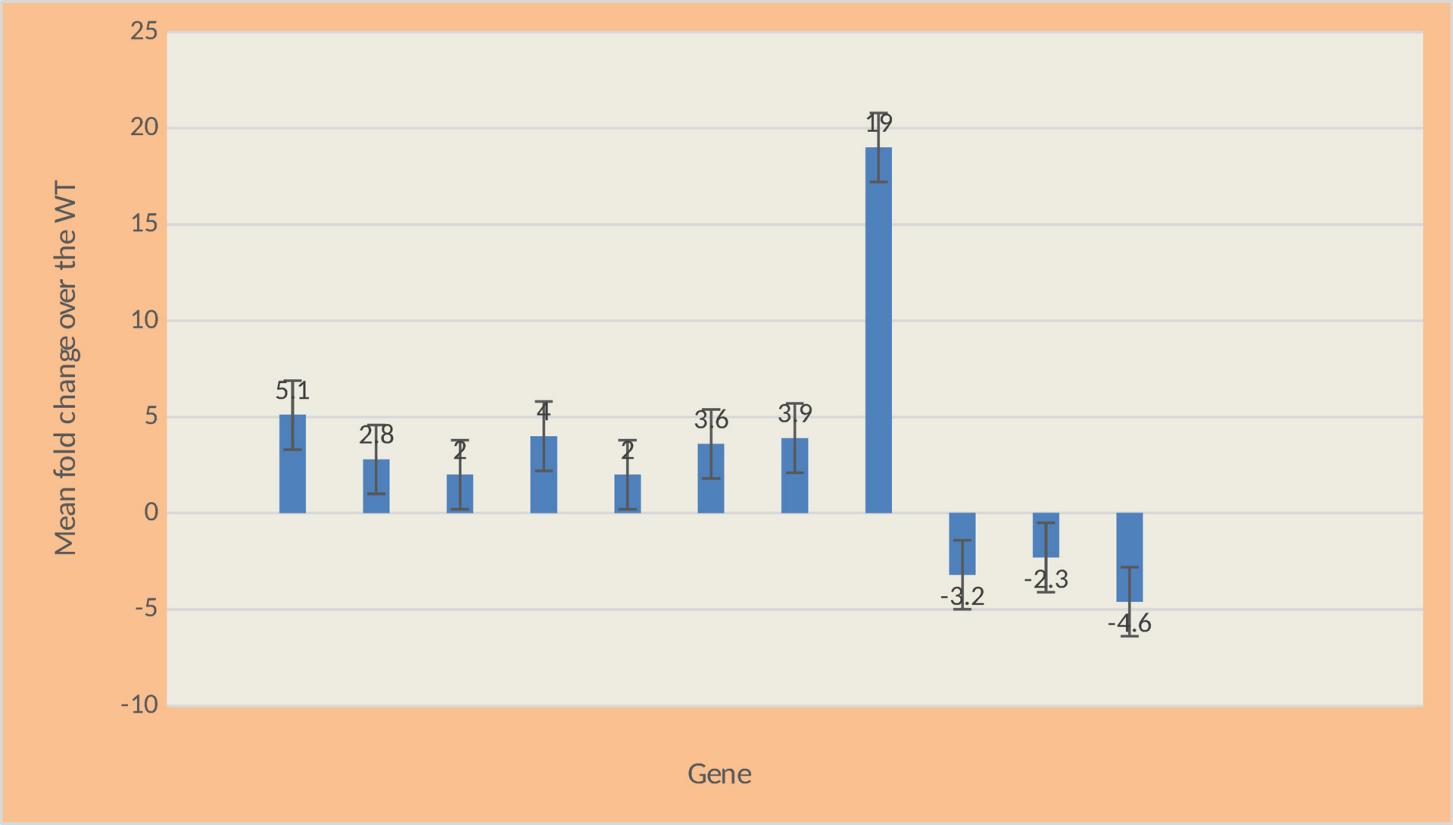
Real time RT-PCR analysis of selected genes.

After the concentration of the RNA was established, the protocol from the Bio-Rad iTaq^TM^ Universal SYBR^®^ Green One-Step Kit was followed with some modifications to perform the expression analysis on selected genes. Please refer to Table 2 for gene names and primers. The genes that were analyzed for differential expression by using real time RT-PCR were *fnr*, *fecR*, *cfa*, *wcaC*, *wcaK*, *pykF*, *pgi*, *pgk*, *acnB*, *sdhC*, *cyoA*. (Fig 9).

### Glycolysis genes

1. Expression of the *pgi* gene is 19-fold higher in the mutant cells than in the WT cells.
2. Expression of the *pykF* gene is 3.6-fold higher in the mutant cells than in the WT cells.
3. Expression of the *pgk* gene is 3.9-fold higher in the mutant cells than in the WT cells.

### TCA cycle and electron transport genes

1. Expression of the *acnB* gene is 3.2-fold lower in the mutant cells than in the WT cells.
2. Expression of the *sdhC* gene is 2.3-fold lower in the mutant cells than in the WT cells.
3. Expression of the *cyoA* gene is 4.6-fold lower in the mutant cells than the WT cells.

### Biofilm genes

1. Expression of the *wcaC* gene is 4.0-fold higher in the mutant cells than the WT cells.
2. Expression of the *wcaK* gene is 2.0-fold higher in the mutant cells than the WT cells.

### Iron uptake and cyclopropane fatty acid synthase genes

1. Expression of the *fecR* gene is 2.8-fold higher in the mutant cells than the WT cells.
2. Expression of the *cfa* gene is 2.0-fold higher in the mutant cells than the WT cells.

### Fumarate nitrate reductase gene

1. Expression of the *fnr* gene is 5.1-fold higher in the mutant cells than the WT cells.

## Discussion

The presence of small colony variants has been known for over a century, but it was in the last twenty years that more serious investigation of this type of strain was undertaken with *Staphylococcus aureus* [1, 5]. This research was stimulated by the observation that persistent and relapsing infections were associated with this microorganism [7]. *S*. *aureus* SCVs show typical behavior in that they are auxotrophs and grow slowly even in rich medium. For *S*. *aureus*, SCVs commonly found were deficient for menadione or hemin, which are required for the proper synthesis of cytochromes and hence fully functional electron transport [1]. Thus the SCVs have insufficient ATP to support normal growth. However, if the SCVs are supplemented with the missing metabolite, their growth rate is restored to normal, and their defective phenotypes are rescued [1].

Another type of SCV resulted from thymidine auxotrophy. Initially, this type of SCV was shown to have similarities to the electron transport type of SCV. Eventually, thymidine insufficiency was shown to act more indirectly on electron transport [8,15], as it has diminished TCA cycle activity, which in turn results in down regulation of electron transport [8,15]. Extensive investigation by a proteomic analysis of a representative SCV of the *S*. *aureus* electron transport class (*hemB* mutation) showed that it displayed upregulation of glycolysis proteins, such as, glyceraldehyde-3-phosphate dehydrogenase and phosphoglycerate kinase. Moreover, transcriptional analysis of a TCA cycle enzyme aconitase indicated down regulation of the cycle [16]. A more recent study indicates that reduced TCA cycle activity is a common feature of *S*. *aureus* SCVs [17]. As stated above, addition of the auxotrophic metabolite results in reversal of the SCV phenotypes [1].

The data presented in this study on an *E*. *coli* SCV are parallel to those with *S*. *aureus*. In this case the auxotrophic molecule is lipoic acid, which is a necessary cofactor for the functioning of pyruvate dehydrogenase and alpha-ketoglutarate dehydrogenase [14]. If these two enzymes are not functioning well, this will cripple the TCA cycle and lead to depressed electron transport, and ultimately low ATP. qRT-PCR demonstrates that as with the *S*. *aureus* electron transport defective SCVs, the *E*. *coli* SCV has an increase in expression of glycolysis genes, e.g., phosphoglucose isomerase and pyruvate kinase. Additionally, there was a depression of gene expression for TCA cycle genes, aconitase and succinate dehydrogenase, and for electron transport as cytochrome o oxidase expression was markedly reduced.

Like *S*. *aureus* SCVs, addition of a sufficient concentration of the auxotrophic molecule (5 μg/ml of lipoic acid) resulted in a phenotypic rescue of morphological, physiological and molecular markers in log phase cells. Morphologically the SCV changed from a small, smooth, convex, glossy appearance to the beige, irregular presentation of the WT morphology. The SCV became sensitive to acid and hydrogen peroxide like the WT strain. And at the molecular level it was demonstrated by RT-PCR that the expression of the phosphoglycerate kinase gene and the colanic acid biosynthesis gene *wcaK* were reduced to the lower WT level.

What appears to be a unique aspect of this study is that the *lipA* SCV is resistant to hostile environments in log phase without pre-exposure. So the strain can withstand acid, hydrogen peroxide, heat and osmotic shock. At least a partial explanation for this consistent result is the increase in the cyclopropane fatty acids (CFA) composition of this strain in log phase. In conjunction with this observation, the expression of the CFA synthase is elevated in log phase cells. This enzyme converts membrane bound *cis*-monounsaturated fatty acids C16:1 and C18:1 to the respective *cis*- C17 and C19 CFAs using methyl groups from S-adenosylmethionine as the donor [18]. It has already been established that CFAs can confer acid resistance to *E*. *coli* [19–21], and there is also evidence that CFA can protect against high temperature [21]. To give perspective, the maximal production of CFAs has been shown to occur in stationary phase [20], and in the lipA SCV this membrane component accounts for about 40% of the whole cell lipid compared to 20% for the WT. So the 3-fold increase in CFA in *lipA* in log phase is not maximal and it is likely that other factors play a role in the log-phase resistance demonstrated by this strain. It has also been noted that slow-growing bacterial cells have elevated levels of CFA [22].

When high *fnr* (ferric nitrate reductase) expression in *lipA* was first observed, it was at first puzzling. Why should a gene that is an important regulator under anaerobic conditions [23–24] be expressed so well in cells that are grown aerobically? This issue is addressed in detail by Kohler et al. who employed both two-dimensional gel electrophoresis for proteomics, and an extensive DNA microarray for transcriptomics to analyze expression in *S*. *aureus menD* and *hemB* SCVs [25]. An overview of their work indicates that although the *menD* or *hemB* mutants were grown aerobically their gene expression was similar to that of a WT strain grown anaerobically. For example, glycolytic and fermentation proteins increased in amount, and TCA proteins declined significantly in amount. We report similar results. With the *lipA* mutant global changes in gene expression occur when sufficient lipoic acid is added to the LB medium, and a WT pattern emerges. The same is true for the *S*. *aureus* SCV model and menadione.

From an evolutionary perspective, the *lipA* SCV represents a form of *E*. *coli* that has survival capacity. As has been stated, *S*. *aureus* SCVs represent a sub-population of cells that have strong persister capacity [1, 5]. The *lipA* SCV would not be expected to compete with its wild type counterpart in an environment that is nutritionally rich such as LB, but apparently almost devoid of lipoic acid. If the *lipA* SCV is grown under this circumstance, it expresses genes that prime it for survival as witnessed by its ability to robustly survive hostile environments such as lethal acid, a lethal concentration of hydrogen peroxide and a temperature that is much more destructive to its wild type counterpart. If lipoic acid is added then the SCV goes through a global reprogramming of gene expression and grows as well as the wild type, but it loses its persistence capacity. The fact that colanic acid biosynthesis genes such as *wcaC* and *wcaK* are upregulated in the SCV suggests that the strain may have an improved capacity for forming a biofilm, and this type of structure has been suggested to be a means of bacterial persistence [26].

## Conclusions

The real time RT-PCR results indicate that the genes *fnr*, *fecR*, *wcaC*, *wcaK* and *cfa* are upregulated in the *lipA* SCV, and may be biomarkers for *E*. *coli* SCVs. Furthermore, the *E*. *coli* strain *lipA* has the properties of a small colony variant, and is similar in its gene expression patterns to the electron transport defective SCVs of *S*. *aureus*. The *E*. *coli* SCV is very resilient in its ability to endure severe stress in log phase, such as pH 3 or hydrogen peroxide or high temperature. Cyclopropane fatty acids may play at least a partial role in this ability to survive.

## Supporting Information

S1 Fig Gel electrophoresis comparing expression of glycolysis genes (*eno*), (*fbaA*), and (*pgi*) in WT and *lipA*. Lane 1 Molecular markers; Lane 2 WT Enolase; Lane 3 *lipA* Enolase; Lane 4 WT Aldolase; Lane 5 *lipA* Aldolase; Lane 6 WT Glucosephosphate isomerase; Lane 7 *lipA* Glucosephosphate isomerase
(TIFF)

S2 Fig Gel electrophoresis comparing expression of glycolysis genes (*pgk*) and (*pykF*) in the WT and *lipA*. Lane 1 Molecular markers; Lane 2 WT Phosphoglycerate kinase; Lane 3 *lipA* Phosphoglycerate kinase; Lane 4 WT Pyruvate kinase; Lane 5 *lipA* Pyruvate kinase
(TIFF)

S3 Fig Gel electrophoresis comparing glycolysis genes (*pykF) and (pgk*) in WT, *lipA*, *lipA* / lipoic acid. Lane 1 molecular markers; Lane 2 WT, *pykF* gene; Lane 3 mutant, *pykF* gene; Lane 4 mutant grown in lipoic acid, *pykF* gene; Lane 5 WT, *pgk* gene; Lane 6 mutant *pgk* gene; Lane 7 mutant grown in lipoic acid, *pgk* gene
(TIFF)

S4 Fig Gel electrophoresis comparing biofilm/colanic acid genes *wcaC* and *wcaK* in the WT, and *lipA*. Lane 1 molecular markers; Lane 2 WT *wcaC;* Lane 3 mutant *wcaC;* Lane 4 WT *wcaK;* Lane 5 mutant *wcaK*
(TIFF)

S5 Fig Gel electrophoresis comparing *wcaK* in the WT, *lipA* and *lipA* strain with 5μg/ml lipoic acid. Lane 1 WT *wcaK* gene; Lane 2 *lipA wcaK* gene; Lane 3 *lipA* grown in lipoic acid *wcaK* gene
(TIFF)

S6 Fig Gel electrophoresis comparing *wcaC* in the WT, *lipA*, and *lipA* strain supplemented with lipoic acid. Lane 1 molecular markers; Lane 2 WT, *wcaC* gene; Lane 3 mutant, *wcaC* gene; Lane 4 mutant strain grown LB containing 5μg/ml lipoic acid, *wcaC* gene
(TIFF)
