# Supplementary figures and images for: The Physiological and Molecular Characterization of a Small Colony Variant of Escherichia coli and Its Phenotypic Rescue
Source: PLoS One. 2016 Jun 16;11(6):e0157578. doi: 10.1371/journal.pone.0157578 (PMC4910995; doi:10.1371/journal.pone.0157578)

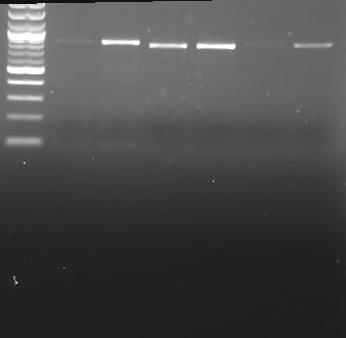

Supplement: S1 Fig — Lane 1 Molecular markers; Lane 2 WT Enolase; Lane 3 lipA Enolase; Lane 4 WT Aldolase; Lane 5 lipA Aldolase; Lane 6 WT Glucosephosphate isomerase; Lane 7 lipA Glucosephosphate isomerase (TIFF) [file pone.0157578.s001.tiff]

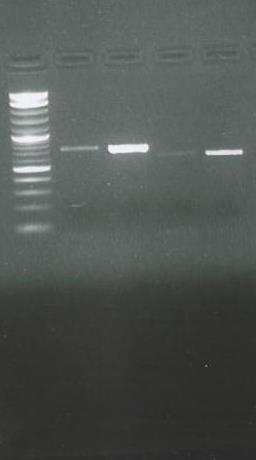

Supplement: S2 Fig — Lane 1 Molecular markers; Lane 2 WT Phosphoglycerate kinase; Lane 3 lipA Phosphoglycerate kinase; Lane 4 WT Pyruvate kinase; Lane 5 lipA Pyruvate kinase (TIFF) [file pone.0157578.s002.tiff]

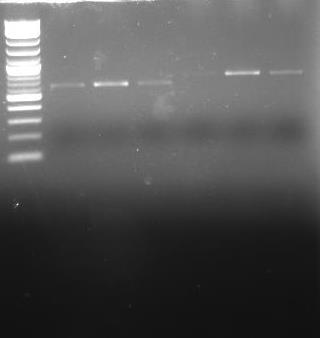

Supplement: S3 Fig — Lane 1 molecular markers; Lane 2 WT, pykF gene; Lane 3 mutant, pykF gene; Lane 4 mutant grown in lipoic acid, pykF gene; Lane 5 WT, pgk gene; Lane 6 mutant pgk gene; Lane 7 mutant grown in lipoic acid, pgk gene (TIFF) [file pone.0157578.s003.tiff]

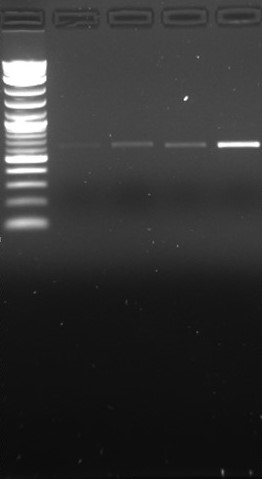

Supplement: S4 Fig — Lane 1 molecular markers; Lane 2 WT wcaC; Lane 3 mutant wcaC; Lane 4 WT wcaK; Lane 5 mutant wcaK (TIFF) [file pone.0157578.s004.tiff]

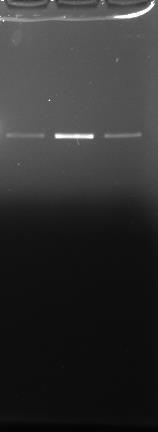

Supplement: S5 Fig — Lane 1 WT wcaK gene; Lane 2 lipA wcaK gene; Lane 3 lipA grown in lipoic acid wcaK gene (TIFF) [file pone.0157578.s005.tiff]

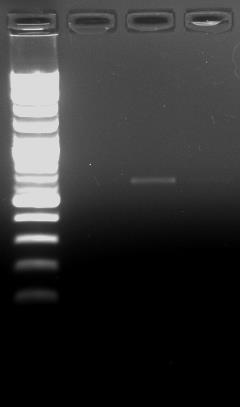

Supplement: S6 Fig — Lane 1 molecular markers; Lane 2 WT, wcaC gene; Lane 3 mutant, wcaC gene; Lane 4 mutant strain grown LB containing 5μg/ml lipoic acid, wcaC gene (TIFF) [file pone.0157578.s006.tiff]
